# Immunization with the HisAK70 DNA Vaccine Induces Resistance against *Leishmania Amazonensis* Infection in BALB/c Mice

**DOI:** 10.3390/vaccines7040183

**Published:** 2019-11-14

**Authors:** Abel Martínez-Rodrigo, Daniel S. Dias, Patrícia A. F. Ribeiro, Bruno M. Roatt, Alicia Mas, Javier Carrión, Eduardo A. F. Coelho, Gustavo Domínguez-Bernal

**Affiliations:** 1 Departamento de Sanidad Animal, Facultad de Veterinaria, Universidad Complutense Madrid, INMIVET, 28040 Madrid, Spain; abelma01@ucm.es (A.M.-R.); alimas@ucm.es (A.M.); javier.carrion@ucm.es (J.C.); 2 Programa de Pós-Graduação em Ciências da Saúde: Infectologia e Medicina Tropical, Faculdade de Medicina, Universidade Federal de Minas Gerais, Belo Horizonte 30130-100, Minas Gerais, Brazil; daniel-sdias@hotmail.com (D.S.D.); patty-fernandes@hotmail.com (P.A.F.R.); dudaferc@ufmg.br (E.A.F.C.); 3 Laboratório de Imunopatologia, Núcleo de Pesquisas em Ciências Biológicas, Universidade Federal de Ouro Preto, Ouro Preto 35400-000, Minas Gerais, Brazil; roatt@ufop.edu.br; 4 Departamento de Patologia Clínica, COLTEC, Universidade Federal de Minas Gerais, Belo Horizonte 31270-901, Minas Gerais, Brazil

**Keywords:** *Leishmania amazonensis*, cross-protection, leishmaniosis, vaccine, DNA, HisAK70

## Abstract

*Leishmania amazonensis* is the aetiological agent of a broad spectrum of leishmaniosis in South America. It can cause not only numerous cases of cutaneous leishmaniosis but also diffuse cutaneous leishmaniosis. Considering the diversity of parasite species causing different forms of the disease that coexist in the same region, it is desirable to develop a vaccine capable of eliciting cross-protection. We have previously described the use of HisAK70 DNA vaccine for immunization of mice to assess the induction of a resistant phenotype against *Leishmania major* and *infantum* infections. In this study, we extended its application in the murine model of infection by using *L. amazonensis* promastigotes. Our data revealed that 14 weeks post-infection, HisAK70-vaccinated mice showed key biomarkers of protection, such as higher iNOS/arginase activity, IFN-γ/IL-10, IFN-γ/IL-4, and GM-CSF/IL-10 ratios, in addition to an IgG2a-type response when compared to the control group. These findings correlated with the presentation of lower footpad swelling and parasite burdens in the immunized compared to the control mice. Overall, this study suggests that immunization with HisAK70 may be considered a suitable tool to combat leishmaniosis as it is able to induce a potent cellular immune response, which allows to control the infection caused by *L. amazonensis*.

## 1. Introduction

Leishmaniosis encompasses a group of parasitic diseases caused by infection with different species of the intracellular kinetoplastid protozoan of the genus *Leishmania* [1]. Leishmaniosis is a vector-borne disease considered endemic in distinct areas of the tropics, subtropics and the Mediterranean Basin, including 97 countries worldwide, with a total of 350 million people at risk of contracting the infection [2]. Although it has a long history [1], leishmaniosis still ranks in the top three neglected tropical diseases (NTD) caused by protozoa [3], being the second most common cause of death among tropical infections [4]. Despite being an NTD, a number of studies on leishmaniosis have been published recently, which have shown how the parasites are adapting to changing environments and spreading into new geographical areas worldwide [5,6,7].

There are four main forms of the disease, visceral leishmaniosis or kala-azar (VL), which is lethal if acute and left untreated; post-kala-azar dermal leishmaniosis (PKDL); cutaneous leishmaniasis (CL) and mucocutaneous leishmaniasis (MCL). While VL is more severe, CL is the most common form of the disease [8] and is characterized by skin lesions leaving life-long scars and disabilities, thus resulting in a great social stigma and causing morbidity. *Leishmania amazonensis* is among the species causing CL and ML in South America. Brazil is among the 10 countries with the highest incidence of CL that together account for 70 to 75% of the estimated global occurrence [9]. This issue is becoming even more relevant, as recent findings also showed that *L. amazonensis* is spreading into new transmission areas in Brazil, leading to unusual clinical manifestations, such as VL and diffuse cutaneous leishmaniosis [10,11], and even causing the first described case of VL in a dog [12].

The control of *L. amazonensis* infection is complex, mainly because the natural transmission of this agent depends on the sandfly vector *Lutzomyia flaviscutellata*, and its maintenance relies on several small wild animal hosts, especially marsupials and rodents such as spiny rats from the genus *Proechimys* spp. [13]. Disease control depends on the early diagnosis and treatment of active cases, although it is widely accepted that a prophylactic vaccine for human leishmaniosis is the way to successfully eliminate the disease. Furthermore, there is currently no acceptable vaccine for use in humans to prevent leishmaniosis [14], and conventional chemotherapies for the treatment of the disease are usually long and not effective due to the toxicity and the presence of resistance parasites [15,16]. A novel immunochemotherapy method based on *L. amazonensis* lysate has been recently licensed in Brazil [17]. In addition, access to treatment in poor countries with high parasite burden is challenging. New approaches for both nanomedicine and treatment at the local site of infection are needed to reduce the toxicity and increase the accessibility of treatments [15,16,18].

In this context, vaccinations seem to be the best option to control leishmaniases [19], as patients who recover from the disease develop long-lasting immunity to subsequent infections against the same species but also against other species [20]. The induction, in the absence of side effects, of long-term cross-protection is an important requirement for the development of more effective *Leishmania* vaccines, consequently, some vaccine candidates are being tested against different *Leishmania spp*. [21,22,23]. An effective vaccine must also induce the development of an antiparasitic CD4^+^ and CD8^+^ T-cell-mediated Th1 immunity, which is characterized by the production of interferon-γ (IFN-γ), interleukin-2 (IL-2) and IL-12, among other pro-inflammatory cytokines [24,25]. On the other hand, susceptible murine models, such as the BALB/c mice model of CL induced by *L. amazonensis*, are associated with the development of a Th2-type response, with high levels of parasite-specific induction of IL-4 and IL-10 along with elevated antileishmanial immunoglobulin G1 (IgG1) antibody production [26,27,28,29].

With the aim of fulfilling these features, we explored suitable prophylactic strategies for the development of a vaccine against multiple *Leishmania* species using efficient multi-antigen formulations. It has been previously described that HisAK70, a DNA vaccine candidate encoding seven *Leishmania* genes (H2A, H2B, H3, H4, A2, KMP11 and HSP70) with broad species specificity, induced and effective cross-protective immunity in mice against *L. infantum* and *L. major* infections [21,30]. Recently, a vaccination approach that exploits an attenuated mutant of *Salmonella enterica* serovar Choleraesuis as a carrier to deliver a plasmid encoding the HisAK70 protein has been published as an alternative way to induce a resistant phenotype against murine VL [31]. In the present study, taking another step towards achieving global control of leishmaniosis, we explore the cross-protection potential of HisAK70 as a DNA vaccine administered in a known murine model against *L. amazonensis* challenge [26,32].

## 2. Materials and Methods

### 2.1. Mice, Parasites and Preparation of Soluble Ag

Eight-week-old female BALB/c mice were obtained from the breeding facilities of the Department of Biochemistry and Immunology, Institute of Biological Sciences, UFMG (Brazil) and were maintained under specific-pathogen-free conditions. *L. amazonensis* (IFLA/BR/1967/PH-8) was cultured as previously described [33]. The soluble *L. amazonensis* antigen (SLA) was prepared from stationary-phase promastigotes as described elsewhere [34]. The study was approved by the Committee on the Ethical Handling of Research Animal of UFMG (code number 333/2015).

### 2.2. Vaccine Preparation and Immunization Protocol

The DNA vaccine pVAX1:HisAK70-asd (HisAK70) and the empty vector pVAX1-asd (pVAX) were previously constructed as described [21]. Briefly, the DNA plasmids were purified using the EndoFree Plasmid Giga Kit (Qiagen, Hilden, Germany) according to the manufacturer’s instructions. The endotoxin-free DNA plasmids were resuspended in sterile saline solution and stored at −20 °C until use. Two groups of mice (*n* = 20) were subcutaneously (s.c.) immunized with 175 µg of HisAK70 (HisAK70) or pVAX (vector) diluted in 50 µL saline in their left hind footpad on days 60, 45 and 30. In parallel, a group of control mice (*n* = 10) was inoculated with 50 µL of sterile saline by the same procedure.

### 2.3. Generation of Bone Marrow-Derived Murine Dendritic Cells (BMDCs) for Use in Pre/post-Infection Assays

Bone marrow stem cell progenitors were obtained from the femurs and tibiae of naïve BALB/c mice (*n* = 3) and cultured in completed medium (CM) consisting of RPMI (1640 with L-Glutamine, Lonza, Basel, Switzerland), supplemented with 10 % FBS (Gibco, Life Technologies, Thermo Fisher Scientific, Waltham, MA, USA) and a mixture of antibiotics (100 U/mL penicillin, 100 mg/mL streptomycin, Lonza) and 10 mM of HEPES (Lonza) with the presence of 20 ng/mL murine granulocyte macrophage colony-stimulating factor (GM-CSF; PeproTech, London, UK), as previously described [35]. Fresh medium containing GM-CSF was added to the cultures every 3 days, and on day 7, the non-adherent cells were collected and considered BMDCs based on the expression of CD11c as described elsewhere [31].

### 2.4. Pre-Infection Evaluation of the Immune Response Induced by HisAK70 Immunization

The immunization efficacy before the in vivo challenge was analysed by measuring the ability of lymphocytes from vaccinated mice to confer killing activity to naïve BMDCs. Briefly, five mice per group were euthanized, the spleens were collected, and naïve BMDCs were obtained as described [5,21,31]. After 7 days of differentiation, the cells were seeded (5 × 10^5^ cells/mL) into 24-well plates with polylysine-treated coverslips (13 mm diameter, VWR) and cultured overnight. On the following day, stationary-phase *L. amazonensis* promastigotes were added at a 5:1 ratio (parasites:BMDCs). After 4 h of incubation at 37 °C, extracellular parasites were removed by washing, and cells were incubated for 24 and 72 h in the presence of splenocytes from the immunized mice at a ratio of 1:5 (BMDCs:splenocytes). After Giemsa staining, cells were mounted with Coverquick 3000 (Labonord, Templemars, France), and 400 cells were counted using an Olympus BX41 microscope. The percentage of infected cells and the mean number of intracellular amastigotes per infected cell were evaluated. The infection index was calculated by multiplying both parameters to account for the overall parasite load [5].

To observe changes in the cytokine levels induced by immunization, naïve BMDCs were seeded (5 × 10^5^ cells/mL) into 24-well plates and pulsed or not with 25 µg/mL SLA overnight. Subsequently, BMDCs were incubated in the presence of splenocytes from the immunized and euthanized mice (*n* = 5 each group) at a 1:5 ratio (BMDCs:splenocytes). Supernatants were collected after 96 h, and IFN-γ, IL-10, IL-4 and GMCSF levels were measured by using commercial ELISA kits following the manufacturer’s instructions (Duoset ELISA, Development System R&D, Abingdon, UK).

### 2.5. Infection, Lesion Follow-up and Parasite Burden in the In Vivo Model

To evaluate the immunization efficacy against CL, 30 days after the last immunization, mice (*n* = 5 per group) were infected subcutaneously in their right footpad with 10^6^ *L. amazonensis* stationary promastigotes. The course of infection was monitored weekly by measuring footpad swelling thickness with a metric calliper and was expressed as the increase in thickness of the infected footpad compared to the uninfected footpad. Fourteen weeks post-infection, animals were euthanized, and sera, lesion fragment, spleen, liver and popliteal draining lymph nodes (dLn) were collected for parasitological and immunological analysis. To evaluate the parasite load, infected tissues were subjected to a limiting dilution assay as already described [36]. Briefly, tissues were homogenized using a glass tissue-grinder in 2 mL of complete Schneider’s medium. Two hundred microliters were plated into 96-well flat-bottom microtiter plates (Nunc, Nunclon) and diluted in log-fold serial dilutions from 10^−1^ to 10^−12^ using complete Schneider’s medium. Each sample was plated in quadruplicate and maintained at 24 °C until read (10 days). The results were expressed as the log of the number of parasites in each organ calculated from the reciprocal of the highest dilution containing viable promastigotes.

### 2.6. Cellular Immune Response in the Spleen: Cytokine Production after Challenge

After 14 weeks of infection, a co-culture system of naïve BMDCs and splenocytes from immunized and infected mice was performed as described previously to analyse the immunological response of vaccinated animals against infection. Briefly, BMDCs were seeded (5 × 10^5^ cells/mL) into 24-well plates and pulsed or not with 25 µg/mL SLA overnight. Once the spleens were collected, splenocytes were obtained and added into the co-culture system at a 1:5 ratio (BMDCs:splenocytes). The supernatants were collected after 96 h, and IFN-γ, IL-10, IL-4, IL-12 and GM-CSF levels were measured by using commercial ELISA kits following the manufacturer’s instructions (Duoset^®^ ELISA, Development System R&D, Abingdon, UK).

In parallel, the cell response was also evaluated by flow cytometry. For this purpose, spleen cells (5 × 10^6^ cells) from immunized and infected animals were collected and stimulated in vitro in polypropylene tubes (Becton Dickinson, Falcon Franklin Lakes, NJ, USA) with SLA (25 μg/mL) for 48 h at 37 °C in 5% CO_2_, whereas the non-stimulated culture received only completed medium. The intracytoplasmic IFN-γ-, TNF-α-, and IL-10-producing T-cell profile was measured as described elsewhere [37]. Briefly, the measurements were performed on a FACSCalibur^®^ instrument, and the Cell-Quest™ software package (Becton Dickinson, Franklin Lakes, NJ, USA) was used for analysis based on 30,000 events per sample. Density plot distribution graphs of CD4^+^/FL1 or CD8^+^/FL1 versus IFN-γ/FL-2, TNFα/FL-2, or IL-10/FL2 were constructed to determine the percentage of IFN-γ^+^, TNF-α^+^, and IL-10^+^ T cells. The results were expressed as indexes that were obtained by the ratio of the percentage of CD4^+^ and CD8^+^ T cells in the SLA-stimulated cultures to the values obtained for the non-stimulated cells (ratio: stimulated culture/non-stimulated culture).

### 2.7. Arginase Activity and Nitric Oxide Production Assay

The concentration of nitrites, which are a by-product of nitric oxide (NO) production, was measured in the supernatant from the co-culture system (splenocytes:SLA-pulsed BMDCs) after 96 h using Griess reaction as described [38]. Subsequently, the cells were incubated for 30 min in lysis buffer (0.1 M Tris–HCl, pH 7.5, 300 μM NaCl, 1 μM PMSF, 1% Triton X-100), and lysates were assayed for intracellular arginase activity as previously described [39]. One unit of enzyme activity was defined as the amount of enzyme that catalyses the formation of 1 mmol of urea/min.

### 2.8. Humoral Response

Blood samples were collected from the animals 30 days after immunization and 14 weeks after infection (*n* = 5 animals for each group and time point). Standard endpoint ELISA was performed as previously described [31] to determine both anti-SLA and anti-poly-protein cocktail HisAK70 antibodies (Abs) at 30 days post-immunization and anti-SLA Abs at 14 weeks after infection. Briefly, 96-well flat-bottomed microtiter plates (Nunc Immunoplate, Maxisorb) were coated overnight at 4 °C with 100 μL of SLA (25 μg/mL) or each peptide of the HisAK70 cocktail (10 μg/mL of four histone peptides, 10 μg/mL of A2 peptides, 10 μg/mL of Kmp11, or 10 μg/mL of Hsp70) diluted in PBS. Negative and positive control sera were obtained from parasite-free and *L. amazonensis*-infected mice, respectively. Peroxidase-labelled goat anti-mouse IgG (dilution 1/4000, Southern Biotech) and IgG isotypes (IgG1 and IgG2a, dilution 1/10,000, Sigma-Aldrich, St. Louis, MI, USA) were used as secondary Abs. The enzyme-labelled complexes were detected by reaction with the TMB substrate. The reaction was stopped with 50 μL of 2 M sulfuric acid, and the optical density was read using a spectrophotometer at 450 nm.

### 2.9. Statistical Analysis

Data are presented as the mean ± standard deviation (SD) and as the median and the interquartile range in the case of the antibody response. The statistical analyses were performed using GraphPad Prism software (version 6.0 for Windows, San Diego, CA, USA). For normal distribution, analyses were conducted using one-way ANOVA with the multiple range Bonferroni’s test to determine which means from the independent groups (control, vector and HisAK70 groups) were significantly different. The antibody response of animals was analysed using Kruskal-Wallis’s test, as these data do not follow a normal distribution. Differences were considered significant when the *p*-value ≤ 0.05.

## 3. Results

### 3.1. Evaluation or Pre-Infection Biomarkers to Assess the Immune Response Induced by the HisAK70 Vaccine

To elucidate whether immunization with HisAK70 affects the immune response generated in immunized mice, we co-cultured splenocytes from immunized mice with naïve BMDCs. Assessment of the parasite-specific immune response showed that splenocytes from the HisAK70-immunized group produced significantly higher amounts of Th1 cytokines, as shown by the ratios IFN-γ/IL-4, IFN-γ/IL-10, GM-CSF/IL-4 and GM-CSF/IL-10 in comparison to the vector and control groups (Table 1). In addition, the ex-vivo host intracellular arginase activity, an indicator of the susceptibility of infected cells to *Leishmania* parasites, was significantly higher (*p* < 0.05) after 96 h of infection in BMDCs co-cultured with splenocytes from animals of the vector and control groups (50.22 ± 21.03 mU and 63.31 ± 21.34 mU, respectively) than in the HisAK70-vaccinated group (11.27 ± 4.27 mU).

**Figure 1:**
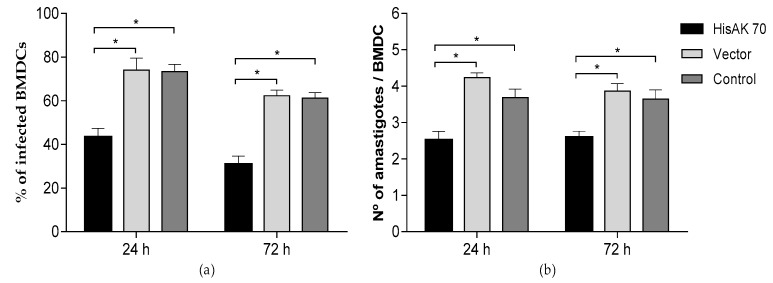
Pre-infection evaluation of the ex-vivo percentage of infected BMDCs (**a**) and number of amastigotes per cell (**b**). Naïve BMDCs infected with *L. amazonensis* were co-cultured with spleen cells from immunized animals, and the above parameters were determined by optical microscopy. Data are presented as the mean ± S.D. Asterisk (*) indicates statistically significant differences between HisAK70 and the vector and control groups (*p* < 0.001).

The immune resistance phenotype was evaluated by the ex-vivo parasite killing activity, where the leishmanicidal potential was investigated by infecting naïve BMDCs and then adding splenocytes from immunized animals. The percentage of infected cells and the number of amastigotes per infected cell in splenocytes from the HisAK70 group were significantly lower (*p* < 0.001), indicating a higher killing potential in BMDCs when compared to those from the vector and control groups at 24 and 72 h post-infection (Figure 1). This fact was substantiated by the percentage of infected cells and the number of amastigotes per infected cell at 24 and 72 h p.i. and the infection index, which combines both parameters (Table 2).

### 3.2. HisAK70 Immunization Confers Protection in Mice Following Challenge with L. amazonensis

**Figure 2:**
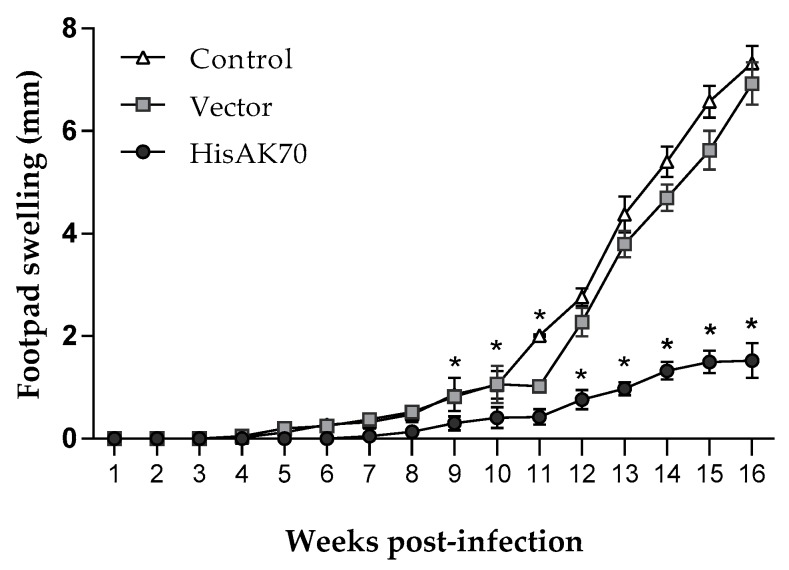
HisAK70 immunization induces protection against *L. amazonensis* infection. Lesion size was measured weekly at the inoculation site during the course of the infection. Data are presented as the mean ± S.D. (*n* = 5). Images macroscopically show the size of the lesion (indicated by arrows) at 12 weeks post-infection. We can observe the differences between immunized and control mice. Asterisk (*) indicates statistically significant differences (* *p* < 0.001) between the HisAK70-vaccinated group and the vector and control groups.

**Figure 3:**
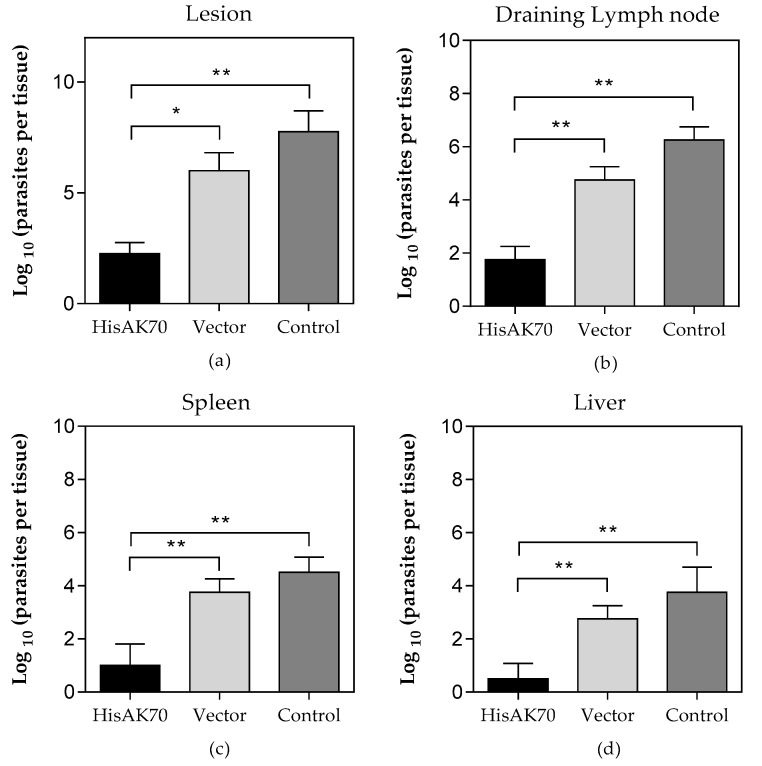
Parasite burden in immunized and infected animals. Fourteen weeks after infection, animals were euthanised, and lesions (**a**), draining lymph-node (**b**), spleen (**c**) and liver (**d**) were used in a limiting dilution assay. Data are presented as the logarithm of the number of parasites per whole organ or tissue ± S.D. (*n* = 5). Asterisks indicate statistically significant differences (*p < 0.05; **p < 0.001) between groups.

With the aim of evaluating whether the ex-vivo immune competence elicited by HisAK70 vaccination could be extended to the *L. amazonensis* challenge, we analysed the progression of footpad swelling once per week over 14 weeks after infection, as well as the parasite burden at the end of the experiment in the lesion fragment, draining lymph node (dLn), spleen and liver. As expected, empty plasmid (vector group) and saline (infection control group) immunizations were ineffective in mice, as noted by the lesion size developed in these animals (Figure 2 and Figure S1). In contrast, we observed that the HisAK70-vaccinated group showed significantly (*p* < 0.05) smaller footpad swelling compared with that in the control groups. This finding correlated with a significantly (*p* < 0.05) lower number of parasites from the HisAK70-immunized animals at the site of infection and the dLn (Figure 3) compared with those in the vector and control groups. Furthermore, the vector and control groups showed a statistically significant (*p* < 0.001) higher parasite visceralization per tissue than that in the HisAK70 group (Figure 3). Overall, the results indicated that HisAK70 immunization in mice promoted protection against *L. amazonensis* infection.

### 3.3. Immunization with HisAK70 Promotes a Predominance of the Cellular Immune Response in Mice after Challenge

**Figure 4:**
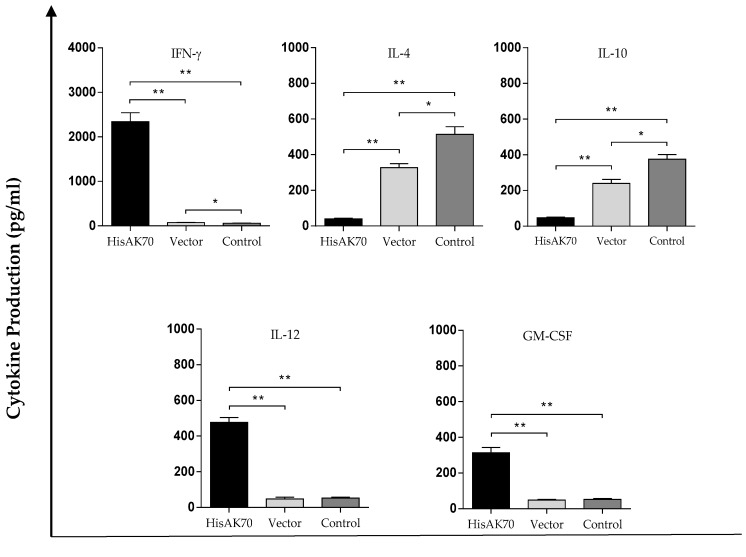
Immunogenicity generated in immunized mice. Fourteen weeks after the infection, animals were euthanised, and splenic cell suspensions were seeded in co-culture with naïve BMDCs, which were previously pulsed with SLA. Cytokine production was measured by ELISA kits. Data are presented as the mean ± S.D. (*n* = 5). Asterisks indicate statistically significant differences (* *p* < 0.05; ** *p* < 0.001) between groups.

In an attempt to determine whether the control of parasite multiplication at the time of infection and the reduction of parasite load in the lesion, dLn, liver and spleen observed in the previous experiment were correlated with the acquisition of a specific cellular immune response, splenic cytokine production was evaluated. As shown in Figure 4, supernatants from co-cultured cells of HisAK70-immunized mice showed significantly higher levels of specific IFN-γ, IL-12 and GM-CSF than those observed in the vector and control groups. In addition, these cells also produced significantly lower amounts of IL-4 and IL-10. These results suggested that HisAK70-immunized mice produced enhanced IFN-γ/IL-4, IFN-γ/IL-10, GM-CSF/IL-4 and GM-CSF/IL-10 ratios compared to those obtained from the vector and control groups (Table 3), which is consistent with the establishment of a protective cellular response.”

**Figure 5:**
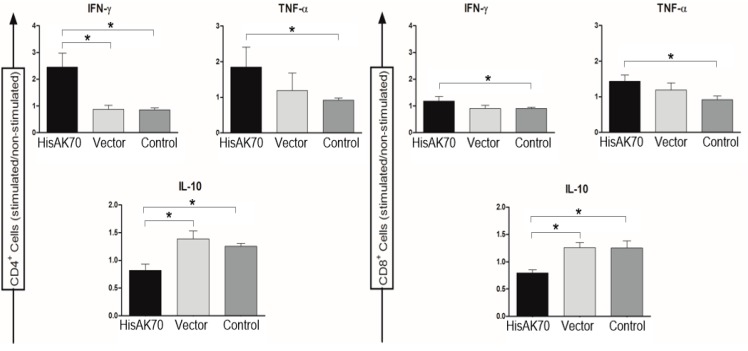
CD^4+^ and CD^8+^ T-cell involvement in the production of cytokines. Fourteen weeks after the infection, animals were euthanized, and spleen cell suspensions were stimulated or not with SLA. The intracytoplasmic production of IFN-γ, TNF-α, and IL-10 by T-cells was measured by flow cytometry. Data are presented as the ratio between stimulated/non-stimulated cells. The asterisks indicate statistically significant differences (* *p* < 0.05) between groups.

Additionally, flow cytometry analyses of splenic cells revealed that the HisAK70 DNA vaccine induced significantly (*p* < 0.05) higher levels of intracytoplasmic IFN-γ- and TNF-α-producing CD4^+^ and CD8^+^ T cell subsets when compared to those in the vector and control groups (Figure 5). Moreover, control mice showed significantly (*p* < 0.05) higher IL-10-producing T-cell indexes. Taken together, the results indicate that HisAK70 vaccination enhances pro-inflammatory/anti-inflammatory cytokine secretion after experimental infection with *L. amazonensis*, thus suggesting a switch from bias to the susceptible *L. amazonensis*-specific Th2 response towards a protective Th1 phenotype.

### 3.4. The Effect of HisAK70 DNA Immunization on Host Enzymatic Activity is Required for Efficient Infection Control

It is well documented that *Leishmania* interacts with the host cell metabolism via arginase or NO synthase [40,41]. Thus, we investigated changes in both enzymatic activities during *L. amazonensis* infection (Table 4). We observed that upregulation of the arginase pathway restricted arginine accessibility to NO synthetase, which resulted in significantly (*p* < 0.001) lower nitrite levels in the vector and control groups. Indeed, HisAK70 immunization enhanced the splenocyte ability to induce NO production in response to DC stimulation with SLA in DC-splenocyte co-culture.

### 3.5. Specific Humoral Response

**Figure 6:**
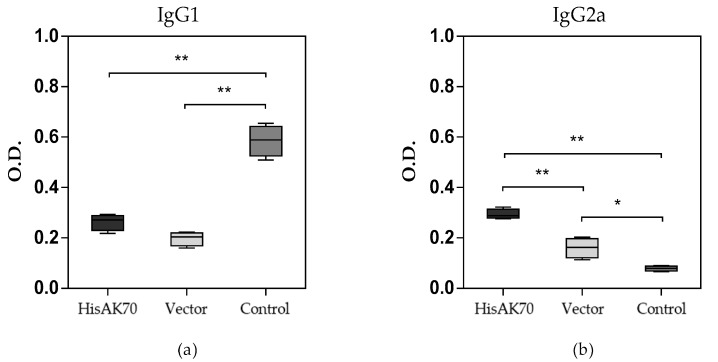
Humoral response after infection. Serum samples were collected from vaccinated and infected mice. The reactivity against SLA was determined by evaluating the IgG1 (**a**) and IgG2a (**b**) isotype antibody levels. The box-and-whisker plots indicate the median and the interquartile range of the optical density values in each group. Asterisks indicate statistically significant differences (* *p* < 0.05; ** *p* < 0.001) between groups.

Since the production of IgG2a antibodies is associated with the establishment of a Th1-type response, whereas the IgG1 subtype is related to a Th2-type response, ELISA assays were performed to quantify the specific anti-SLA and anti-HisAK70 polyprotein humoral responses in the groups. It is noteworthy that all mice (vaccinated and control groups) showed negative anti-SLA or anti-HisAK70 antibody titres after immunization (data not shown), thus confirming the low humoral immunogenicity of the vaccine [42,43]. However, at 14 weeks post infection with *L. amazonensis*, the specific anti-SLA IgG2a response increased significantly in HisAK70-immunized animals, confirming the development of a Th1-biased immune response, which is consistent with the development of a specific antileishmanial response in these animals (Figure 6).

## 4. Discussion

*Leishmania* parasites able to cause CL are usually divided into Old World species (*L. major*, *L. tropica*, and *L. aethiopica*, which are prevalent around the Mediterranean Basin, the Middle East, the Horn of Africa and the Indian subcontinent) and New World species (*L. amazonensis*, *L. mexicana*, *L. braziliensis*, and *L. guyanensis*, which are endemic in the Americas) [44]. Therefore, the development of a single vaccine capable of inducing cross-protection against the different species of the protozoan would be particularly useful in regions where several *Leishmania* species coexist. During the last few years, while a great number of antigens have been examined as vaccine candidates across various *Leishmania* species, only a small number have advanced to human or canine clinical trials [45]. In this context, we have previously demonstrated the features of the HisAK70 DNA vaccine against different forms of leishmaniosis caused by Old World species in the murine model. We have already described that the HisAK70 DNA vaccine encoding seven *Leishmania* antigens (H2A, H2B, H3, H4, A2, KMP11 and HSP70) was able to induce a resistant phenotype against VL and CL caused by *L. infantum* and *L. major*, respectively [21]. Interestingly, these antigens play an essential role in the infectivity stage and other relevant biological features of *Leishmania* [30]. Recently, we evaluated the use of a novel approach that exploits an attenuated mutant of *Salmonella enterica* serovar Choleraesuis as a carrier to deliver the HisAK70 plasmid in a well-described murine model of VL [31]. In addition, we have shown that HisAK70 DNA vaccination followed by an adoptive transfer of DCs pulsed with the HisAK70 polyprotein was successful against an ex-vivo *L. infantum* challenge in dogs, which are considered the main domestic reservoirs in the zoonotic cycle of *L. infantum* transmission [46]. Thus, the main goal of the present study was to evaluate whether immunization with HisAK70 as a DNA vaccine could induce an immune-resistant phenotype against *L. amazonensis* infection, an important causative agent of CL in America. For this reason, we employed the well-characterized BALB/c model of CL by *L. amazonensis* [18,26] in which animals are susceptible to infection and develop chronic lesions [40,47] in the presence of IL-4 and IL-10, in contrast with C57BL/6 mice, where these two Th2-characteristic cytokines seem to have no relevance [24]. With this model, we evaluated the lesion caused by the parasite, as well as the parasite burden in different tissues and organs representing key markers of outcome of infection in an experimental murine model of CL [48]. To further elucidate the immune response produced after immunization with HisAK70, other important biomarkers for resistant and susceptible immune phenotypes, such as cytokine production, arginase activity and iNOS activity, were assessed [31,49,50,51].

In the present study, we evaluated the immune response generated in mice, first after immunization, and then, after the challenge. Predicting the efficacy of HisAK70 immunization before the challenge should be a quality requirement to discontinue further experiments and/or to improve the immunization strategy before subsequent challenges. This evaluation system may also minimize the number of animals used in the experiments, as a non-immunogenic vaccine should not proceed with an in-vivo challenge. With this objective in mind, prior to the challenge, we employed a co-culture system of spleen cells from the immunized animals and naïve BMDCs previously pulsed with SLA or infected with *L. amazonensis* metacyclic promastigotes. Considering that DCs play a central role in initiating a specific T-cell immune response, leading to a resistant immune phenotype in mice, humans and dogs [31,52,53,54,55], together with the importance of memory T cells in establishing an effective long-term immunity [19,52,56,57,58], encouraged us to use this co-culture system. Interestingly, HisAK70 DNA immunization promoted a strong antigen-specific effector Th1 cellular response 30 days post-immunization, as evidenced by higher IFN-γ/IL-10, IFN-γ/IL-4, GMCSF/IL-10 and GMCSF/IL-4 ratios, compared to results obtained in the vector and control mouse groups. In accordance with these data, splenocytes from immunized mice were able to improve *Leishmania* killing activity at 24 and 72 h after ex vivo *L. amazonensis* infection, as reflected by the infection index values (Table 2).

Overall, the effectiveness of the HisAK70 DNA vaccine was based on the ability of immunized mice to achieve the control of key factors such as the production of Th1-characteristic cytokines, factors that indeed are a common feature already described in DNA vaccines [19,59,60].

Subsequently, we evaluated whether the resistant immunophenotype acquired in HisAK70-immunized mice conferred protection against an in-vivo infection using *L. amazonensis* promastigotes. Remarkably, immunization with the HisAK70 DNA vaccine noticeably reduced footpad lesion size when compared to those in the vector and control groups. In addition, HisAK70-immunized mice presented a statistically significant lower parasite burden in all evaluated tissues and organs, namely, the site of infection (lesion), liver, spleen and dLn. It is well established that anti-*Leishmania*-specific immunity is most effectively achieved and maintained over time by the existence of persistent parasites [58]. For that reason, an effective vaccine may require strengthening the immunity against the development of the disease rather than providing sterile protection [61,62]. HisAK70 also favoured an increased iNOS/arginase activity ratio and enhanced antileishmanial immunity based on the levels of Th1-characteristic cytokines such as IFN-γ, IL-12 and GM-CSF, which were statistically significantly higher when compared to those in the vector and control groups. In contrast, the levels of IL-4 and IL-10 cytokines were diminished at 14 weeks post-infection in the HisAK70-immunized animals.

To further investigate the requirements for sustained cellular immunity to an intracellular parasitic infection, we assayed the contribution of CD4^+^ and CD8^+^ T cells to the production of IFN-γ, TNF-α and IL-10 in spleen cells of post-challenged animals [63,64,65,66]. We observed the involvement of CD4^+^ T lymphocyte subset in the higher production of IFN-γ and TNF-α, which was related to the cytokine levels measured in the supernatant of the co-culture system.

Interestingly, our findings showed that there was no generation of specific anti-SLA or anti-HisAK70 polyprotein IgG antibodies after immunization, thus confirming that there was no crossed reactivity with SLA and that there was a low humoral response to the HisAK70 DNA candidate, as previously described [43]. This is a key feature of HisAK70 DNA immunization, mainly because it seems to produce a resistance immune phenotype characterized by a skewed cellular response. Furthermore, as *Leishmania* spp. are intracellular parasites, high levels of anti-SLA antibodies are not effective or desirable. Nevertheless, as observed, after the challenge, there was a production of specific anti-SLA antibodies. In BALB/c mice infected with *L. amazonensis*, the IL-4-dependent production of the IgG1 isotype is associated with disease progression [27,67,68], and the same behaviour is observed in control mice. These data show that non-immunized animals present a susceptible immune phenotype characterized by an inefficient humoral response leading to disease progression, whereas Th1 cytokines from HisAK70-immunized animals together with iNOS and IgG2a production induce a resistant immune phenotype controlling disease progression.

These data indicate that the HisAK70 DNA vaccine, in addition to having an effect against *L. infantum* and *L. major*, can induce cross-protection against species found in the Americas such as *L. amazonensis*. The cross-protection may be due to diverse components in the HisAK70 DNA vaccine such as T cell epitopes with broad species specificity. Our results also indicate that although there is neither absence of lesions nor parasite clearance in parasite target organs, HisAK70 immunization is essential to protect hosts against parasite growth and allow infection control during the late stages of *L. amazonensis* infection. The HisAK70 DNA vaccine continues to be a promising approach against different *Leishmania* spp. and could be well tested as a vaccine candidate to extend its protection to other relevant *Leishmania* species.

## 5. Conclusions

Our data suggest that the HisAK70 DNA vaccine fulfils the requirements for sustained cross-protection of BALB/c mice against *L. amazonensis* infection. HisAK70 was found to induce a sustained specific cellular response as well as low humoral immunity, as confirmed by the acquired killing activity of murine BMDCs in contact with activated splenocytes; the increased NO/ arginase activity ratio; and the appearance of *Leishmania*-specific Th1 cell populations able to produce IFN-γ, IL-12 and GM-CSF upon stimulation with SLA-pulsed BMDCs. The specific cell-mediated immune response against the *L. amazonensis* challenge reduced the footpad swelling and parasite burden and remained effective during the course of infection, probably due to the existence of residual persistent parasites [58,61,62]. Different vaccine delivery systems, such as nanoparticles, should be taken into account for future improvements since they have been demonstrated to not only elicit a strong CD4^+^ and CD8^+^ T cell response [69] but also successfully overcome the persistence parasite presence required for inducing long-term immunity against leishmaniosis, which remains a clear hurdle for accurate vaccine development.

## Figures and Tables

**Table 1:** Ratios of cytokine production from the supernatant of the co-culture system of immunized animals prior to infection.

Groups	Ratio IFN-γ/IL-10	Ratio IFN-γ/IL-4	Ratio GMCSF/IL-10	Ratio GMCSF/IL-4
Control	0.75	3.50	4.02	14.28
Vector	0.47	1.30	3.73	20.87
HisAK70	4.35 *	126.84 *	15.15	428.74 **

Data are presented as the mean ± S.D. Asterisks (*) indicate statistically significant differences (* *p* < 0.05; ** *p* < 0.001) between the HisAK70-vaccinated group and the vector and control groups.

**Table 2:** Evaluation of the infection index obtained by using the percentage of infected cells and the number of amastigotes per infected cell.

Groups	HisAK 70	Vector	Control
24 h after ex vivo infection	10.84 *	31.34	27.09
72 h after ex vivo infection	7.95 *	24.14	22.33

Data are presented as the calculated indexes. Asterisk (*) indicates statistically significant differences (*p* < 0.001) between the HisAK70-vaccinated group and the vector and control groups.

**Table 3:** Ratios of cytokine production from the supernatant of the co-culture system of immunized animals after the challenge.

Groups	Ratio IFN-γ/IL-10	Ratio IFN-γ/IL-4	Ratio GMCSF/IL-10	Ratio GMCSF/IL-4
Control	1.55	0.13	1.47	1.02
Vector	3.12	0.29	2.04	1.49
HisAK70	498.06 **	59.78 **	66.44 *	80.05 **

Data are presented as the mean ± S.D. Asterisks (*) indicate statistically significant differences (* *p* < 0.05; ** *p* < 0.001) between the HisAK70-immunized group and the vector and control groups.

**Table 4:** Arginase metabolism and nitrite determination in mice infected with *L. amazonensis*.

Groups	mU Arginase Activity	µM Nitrites
**Control**	25.32 ± 6.55	0.45 ± 0.13
**Vector**	19.17 ± 5.51	0.78 ± 0.78 *
**HisAK70**	2.61 ± 1.09 **	6.48 ± 0.46 **

Data are presented as the mean ± S.D. Asterisks (**) indicate statistically significant differences (*p* < 0.001) between the HisAK70 vaccinated group and the vector and control groups. Asterisk (*) indicates statistically significant differences (*p* < 0.05) between the vector group and the control group.

## Supplementary Materials

The following are available online at https://www.mdpi.com/2076-393X/7/4/183/s1, Figure S1: HisAK70 immunization induces protection against *L. amazonensis* infection. Lesion size was measured weekly at the inoculation site during the course of the infection. Data are presented as the mean ± S.D. (*n* = 5). Images show macroscopically the size of the lesion (indicated by arrows) at twelve weeks post-infection. We can observe the differences between immunized and control mice.

## References

[1] Akhoundi M., Kuhls K., Cannet A., Votypka J., Marty P., Delaunay P., Sereno D. (2016). A Historical Overview of the Classification, Evolution, and Dispersion of *Leishmania* Parasites and Sandflies. PLoS Negl. Trop. Dis..

[2] World Health Organization Global Health Observatory (GHO) Data. https://www.who.int/gho/neglected_diseases/leishmaniasis/en/.

[3] Fenwick A. (2012). The global burden of neglected tropical diseases. Public Health.

[4] Bern C., Maguire J.H., Alvar J. (2008). Complexities of assessing the disease burden attributable to leishmaniasis. PLoS Negl. Trop. Dis..

[5] Dominguez-Bernal G., Jimenez M., Molina R., Ordonez-Gutierrez L., Martinez-Rodrigo A., Mas A., Cutuli M.T., Carrion J. (2014). Characterisation of the ex vivo virulence of *Leishmania infantum* isolates from *Phlebotomus perniciosus* from an outbreak of human leishmaniosis in Madrid, Spain. Parasites Vectors.

[6] Maroli M., Feliciangeli M.D., Bichaud L., Charrel R.N., Gradoni L. (2013). Phlebotomine sandflies and the spreading of leishmaniases and other diseases of public health concern. Med. Vet. Entomol..

[7] Antoniou M., Gramiccia M., Molina R., Dvorak V., Volf P. (2013). The role of indigenous phlebotomine sandflies and mammals in the spreading of leishmaniasis agents in the Mediterranean region. Euro Surveill. Bull. Eur. Sur Les Mal. Transm. Eur. Commun. Dis. Bull..

[8] World Health Organization (2016). Leishmaniasis in high-burden countries: An epidemiological update based on data reported in 2014. Wkly. Epidemiol. Rec..

[9] Alvar J., Velez I.D., Bern C., Herrero M., Desjeux P., Cano J., Jannin J., den Boer M., WHO Leishmaniasis Control Team (2012). Leishmaniasis worldwide and global estimates of its incidence. PLoS ONE.

[10] Almeida R.P., BarralNetto M., DeJesus A.M.R., DeFreitas L.A.R., Carvalho E.M., Barral A. (1996). Biological behavior of *Leishmania amazonensis* isolated from humans with cutaneous, mucosal, or visceral leishmaniasis in BALB/c mice. Am. J. Trop. Med. Hyg..

[11] Azeredo-Coutinho R.B., Conceicao-Silva F., Schubach A., Cupolillo E., Quintella L.P., Madeira M.F., Pacheco R.S., Valete-Rosalino C.M., Mendonca S.C. (2007). First report of diffuse cutaneous leishmaniasis and *Leishmania amazonensis* infection in Rio de Janeiro State, Brazil. Trans. R. Soc. Trop. Med. Hyg..

[12] Valdivia H.O., Almeida L.V., Roatt B.M., Reis-Cunha J.L., Pereira A.A., Gontijo C., Fujiwara R.T., Reis A.B., Sanders M.J., Cotton J.A. (2017). Comparative genomics of canine-isolated *Leishmania (Leishmania) amazonensis* from an endemic focus of visceral leishmaniasis in Governador Valadares, southeastern Brazil. Sci. Rep..

[13] Osorio y Fortea J., Prina E., de La Llave E., Lecoeur H., Lang T., Milon G. (2007). Unveiling pathways used by *Leishmania amazonensis* amastigotes to subvert macrophage function. Immunol. Rev..

[14] Kumar R., Engwerda C. (2014). Vaccines to prevent leishmaniasis. Clin. Transl. Immunol..

[15] Tavares G.S.V., Mendonca D.V.C., Lage D.P., Antinarelli L.M.R., Soyer T.G., Senna A.J.S., Matos G.F., Dias D.S., Ribeiro P.A.F., Batista J.P.T. (2018). In vitro and in vivo antileishmanial activity of a fluoroquinoline derivate against *Leishmania infantum* and *Leishmania amazonensis* species. Acta Trop..

[16] Mendonca D.V.C., Tavares G.S.V., Lage D.P., Soyer T.G., Carvalho L.M., Dias D.S., Ribeiro P.A.F., Ottoni F.M., Antinarelli L.M.R., Vale D.L. (2019). In vivo antileishmanial efficacy of a naphthoquinone derivate incorporated into a Pluronic^®^ F127-based polymeric micelle system against *Leishmania amazonensis* infection. Biomed. Pharmacother..

[17] Mayrink W., Botelho A.C., Magalhaes P.A., Batista S.M., Lima Ade O., Genaro O., Costa C.A., Melo M.N., Michalick M.S., Williams P. (2006). Immunotherapy, immunochemotherapy and chemotherapy for American cutaneous leishmaniasis treatment. Rev. Soc. Bras. Med. Trop..

[18] Tavares G.S.V., Mendonca D.V.C., Miyazaki C.K., Lage D.P., Soyer T.G., Carvalho L.M., Ottoni F.M., Dias D.S., Ribeiro P.A.F., Antinarelli L.M.R. (2019). A Pluronic^®^ F127-based polymeric micelle system containing an antileishmanial molecule is immunotherapeutic and effective in the treatment against *Leishmania amazonensis* infection. Parasitol. Int..

[19] Iborra S., Solana J.C., Requena J.M., Soto M. (2018). Vaccine candidates against *leishmania* under current research. Expert Rev. Vaccines.

[20] Porrozzi R., Teva A., Amaral V.F., da Costa M.V.S., Grimaldi G. (2004). Cross-immunity experiments between different species or strains of *Leishmania* in rhesus macaques (Macaca mulatta). Am. J. Trop. Med. Hyg..

[21] Dominguez-Bernal G., Horcajo P., Orden J.A., Ruiz-Santa-Quiteria J.A., De La Fuente R., Ordonez-Gutierrez L., Martinez-Rodrigo A., Mas A., Carrion J. (2015). HisAK70: Progress towards a vaccine against different forms of leishmaniosis. Parasites Vectors.

[22] Nico D., Gomes D.C., Alves-Silva M.V., Freitas E.O., Morrot A., Bahia D., Palatnik M., Rodrigues M.M., Palatnik-de-Sousa C.B. (2014). Cross-Protective Immunity to *Leishmania amazonensis* is Mediated by CD4+ and CD8+ Epitopes of *Leishmania donovani* Nucleoside Hydrolase Terminal Domains. Front. Immunol..

[23] Lage D.P., Martins V.T., Duarte M.C., Costa L.E., Tavares G.S.V., Ramos F.F., Chavez-Fumagalli M.A., Menezes-Souza D., Roatt B.M., Tavares C.A.P. (2016). Cross-protective efficacy of Leishmania infantum LiHyD protein against tegumentary leishmaniasis caused by *Leishmania major* and *Leishmania braziliensis* species. Acta Trop..

[24] Ji J., Sun J., Soong L. (2003). Impaired Expression of Inflammatory Cytokines and Chemokines at Early Stages of Infection with *Leishmania amazonensis*. Infect. Immun..

[25] Wanderley J.L.M., Deolindo P., Carlsen E., Portugal A.B., DaMatta R.A., Barcinski M.A., Soong L. (2019). CD4(+) T Cell-Dependent Macrophage Activation Modulates Sustained PS Exposure on Intracellular Amastigotes of *Leishmania amazonensis*. Front. Cell. Infect. Microbiol..

[26] Pereira B.A.S., Alves C.R. (2008). Immunological characteristics of experimental murine infection with *Leishmania (Leishmania) amazonensis*. Vet. Parasitol..

[27] Coelho E.A.F., Tavares C.A.P., Carvalho F.A.A., Chaves K.F., Teixeira K.N., Rodrigues R.C., Charest H., Matlashewski G., Gazzinelli R.T., Fernandes A.P. (2003). Immune responses induced by the *Leishmania (Leishmania) donovani* A2 antigen, but not by the LACK antigen, are protective against experimental *Leishmania* (*Leishmania*) *amazonensis* infection. Infect. Immun..

[28] Ramirez L., Corvo L., Duarte M.C., Chavez-Fumagalli M.A., Valadares D.G., Santos D.M., de Oliveira C.I., Escutia M.R., Alonso C., Bonay P. (2014). Cross-protective effect of a combined L5 plus L3 *Leishmania major* ribosomal protein based vaccine combined with a Th1 adjuvant in murine cutaneous and visceral leishmaniasis. Parasites Vectors.

[29] Campos B.L.S., Silva T.N., Ribeiro S.P., Carvalho K.I.L., Kallas E.G., Laurenti M.D., Passero L.F.D. (2015). Analysis of iron superoxide dismutase-encoding DNA vaccine on the evolution of the Leishmania amazonensis experimental infection. Parasite Immunol..

[30] Dominguez-Bernal G., Horcajo P., Orden J.A., De La Fuente R., Herrero-Gil A., Ordonez-Gutierrez L., Carrion J. (2012). Mitigating an undesirable immune response of inherent susceptibility to cutaneous leishmaniosis in a mouse model: The role of the pathoantigenic HISA70 DNA vaccine. Vet. Res..

[31] Dominguez-Bernal G., Martinez-Rodrigo A., Mas A., Blanco M.M., Orden J.A., De La Fuente R., Carrion J. (2017). Alternative strategy for visceral leishmaniosis control: HisAK70-*Salmonella* Choleraesuis-pulsed dendritic cells. Comp. Immunol. Microbiol. Infect. Dis..

[32] Calabrese K.S., da Costa S.C. (1992). Enhancement of *Leishmania amazonensis* infection in BCG non-responder mice by BCG-antigen specific vaccine. Mem. Inst. Oswaldo Cruz.

[33] Duarte M.C., Lage D.P., Martins V.T., Costa L.E., Carvalho A., Ludolf F., Santos T.T.O., Vale D.L., Roatt B.M., Menezes-Souza D. (2017). A vaccine composed of a hypothetical protein and the eukaryotic initiation factor 5a from *Leishmania braziliensis* cross-protection against *Leishmania amazonensis* infection. Immunobiology.

[34] Scott P., Pearce E., Natovitz P., Sher A. (1987). Vaccination against cutaneous leishmaniasis in a murine model. I. Induction of protective immunity with a soluble extract of promastigotes. J. Immunol..

[35] Lutz M.B., Kukutsch N., Ogilvie A.L., Rossner S., Koch F., Romani N., Schuler G. (1999). An advanced culture method for generating large quantities of highly pure dendritic cells from mouse bone marrow. J. Immunol. Methods.

[36] Buffet P.A., Sulahian A., Garin Y.J.F., Nassar N., Derouin F. (1995). Culture Microtitration—A Sensitive Method for Quantifying *Leishmania-Infantum* in Tissues of Infected Mice. Antimicrob. Agents Chemother..

[37] Duarte M.C., Lage D.P., Martins V.T., Chávez-Fumagalli M.A., Roatt B.M., Menezes-Souza D., Goulart L.R., Soto M., Tavares C.A.P., Coelho E.A.F. (2016). Recent updates and perspectives on approaches for the development of vaccines against visceral leishmaniasis. Rev. Soc. Bras. Med. Trop..

[38] Ding A.H., Nathan C.F., Stuehr D.J. (1988). Release of Reactive Nitrogen Intermediates and Reactive Oxygen Intermediates from Mouse Peritoneal-Macrophages—Comparison of Activating Cytokines and Evidence for Independent Production. J. Immunol..

[39] Garrido V.V., Dulgerian L.R., Stempin C.C., Cerban F.M. (2011). The Increase in Mannose Receptor Recycling Favors Arginase Induction and *Trypanosoma Cruzi* Survival in Macrophages. Int. J. Biol. Sci..

[40] Rath M., Muller I., Kropf P., Closs E.I., Munder M. (2014). Metabolism via arginase or nitric oxide synthase: Two competing arginine pathways in macrophages. Front. Immunol..

[41] Thomas A.C., Mattila J.T. (2014). “Of mice and men”: Arginine metabolism in macrophages. Front. Immunol..

[42] Hasson S.S.A.A., Al-Busaidi J.K.Z., Sallam T.A. (2015). The past, current and future trends in DNA vaccine immunisations. Asian Pac. J. Trop. Biomed..

[43] Kumar A., Samant M. (2016). DNA vaccine against visceral leishmaniasis: A promising approach for prevention and control. Parasite Immunol..

[44] Burza S., Croft S.L., Boelaert M. (2018). Leishmaniasis. Lancet.

[45] Duthie M.S., Reed S.G. (2017). Not All Antigens Are Created Equally: Progress, Challenges, and Lessons Associated with Developing a Vaccine for Leishmaniasis. Clin. Vaccine Immunol..

[46] Martínez-Rodrigo A., Mas A., Fernández-Cotrina J., Belinchón-Lorenzo S., Orden J.A., Arias P., de la Fuente R., Carrión J., Domínguez-Bernal G. (2019). Strength and medium-term impact of HisAK70 immunization in dogs: Vaccine safety and biomarkers of effectiveness for ex vivo *Leishmania infantum* infection. Comp. Immunol. Microbiol. Infect. Dis..

[47] Nascimento K.F., de Santana F.R., da Costa C.R.V., Kaplum V., Volpato H., Nakamura C.V., Bonamin L.V., Buchi D.D. (2017). M1 homeopathic complex trigger effective responses against *Leishmania* (L) *amazonensis* in vivo and in vitro. Cytokine.

[48] Sacks D.L., Melby P.C. (2001). Animal models for the analysis of immune responses to leishmaniasis. Curr. Protoc. Immunol..

[49] Dias D.S., Martins V.T., Ribeiro P.A.F., Ramos F.F., Lage D.P., Tavares G.S.V., Mendonca D.V.C., Chavez-Fumagalli M.A., Oliveira J.S., Silva E.S. (2018). Antigenicity, immunogenicity and protective efficacy of a conserved *Leishmania* hypothetical protein against visceral leishmaniasis. Parasitology.

[50] Dias D.S., Ribeiro P.A.F., Martins V.T., Lage D.P., Ramos F.F., Dias A.L.T., Rodrigues M.R., Portela A.S.B., Costa L.E., Caligiorne R.B. (2018). Recombinant prohibitin protein of *Leishmania infantum* acts as a vaccine candidate and diagnostic marker against visceral leishmaniasis. Cell. Immunol..

[51] Ribeiro P.A.F., Dias D.S., Novais M.V.M., Lage D.P., Tavares G.S.V., Mendonca D.V.C., Oliveira J.S., Chavez-Fumagalli M.A., Roatt B.M., Duarte M.C. (2018). A *Leishmania* hypothetical protein-containing liposome-based formulation is highly immunogenic and induces protection against visceral leishmaniasis. Cytokine.

[52] von Stebut E., Tenzer S. (2017). Cutaneous leishmaniasis: Distinct functions of dendritic cells and macrophages in the interaction of the host immune system with *Leishmania major*. Int. J. Med. Microbiol..

[53] Hosein S., Blake D.P., Solano-Gallego L. (2017). Insights on adaptive and innate immunity in canine leishmaniosis. Parasitology.

[54] Iborra S., Martinez-Lopez M., Cueto F.J., Conde-Garrosa R., Del Fresno C., Izquierdo H.M., Abram C.L., Mori D., Campos-Martin Y., Reguera R.M. (2016). *Leishmania* Uses Mincle to Target an Inhibitory ITAM Signaling Pathway in Dendritic Cells that Dampens Adaptive Immunity to Infection. Immunity.

[55] Freitas-Silva R., Brelaz-de-Castro M.C., Rezende A.M., Pereira V.R. (2014). Targeting Dendritic Cells as a Good Alternative to Combat *Leishmania* spp.. Front. Immunol..

[56] Glennie N.D., Scott P. (2016). Memory T cells in cutaneous leishmaniasis. Cell. Immunol..

[57] Glennie N.D., Volk S.W., Scott P. (2017). Skin-resident CD4+ T cells protect against *Leishmania major* by recruiting and activating inflammatory monocytes. PLoS Pathog..

[58] Scott P., Novais F.O. (2016). Cutaneous leishmaniasis: Immune responses in protection and pathogenesis. Nat. Rev. Immunol..

[59] Seifert K., Juhls C., Salguero F.J., Croft S.L. (2015). Sequential Chemoimmunotherapy of Experimental Visceral Leishmaniasis Using a Single Low Dose of Liposomal Amphotericin B and a Novel DNA Vaccine Candidate. Antimicrob. Agents Chemother..

[60] Viana K.F., Lacerda G., Teixeira N.S., Rodrigues Cangussu A.S., Sousa Aguiar R.W., Giunchetti R.C. (2018). Therapeutic vaccine of killed *Leishmania amazonensis* plus saponin reduced parasite burden in dogs naturally infected with *Leishmania infantum*. Vet. Parasitol..

[61] Sacks D.L. (2014). Vaccines against tropical parasitic diseases: A persisting answer to a persisting problem. Nat. Immunol..

[62] Engwerda C.R., Matlashewski G. (2015). Development of *Leishmania* vaccines in the era of visceral leishmaniasis elimination. Trans. R. Soc. Trop. Med. Hyg..

[63] Gurunathan S., Prussin C., Sacks D.L., Seder R.A. (1998). Vaccine requirements for sustained cellular immunity to an intracellular parasitic infection. Nat. Med..

[64] Perez-Jimenez E., Kochan G., Gherardi M.M., Esteban M. (2006). MVA-LACK as a safe and efficient vector for vaccination against leishmaniasis. Microbes Infect..

[65] Lage D.P., Martins V.T., Duarte M.C., Garde E., Chavez-Fumagalli M.A., Menezes-Souza D., Roatt B.M., Tavares C.A.P., Soto M., Coelho E.A.F. (2015). Prophylactic properties of a *Leishmania*-specific hypothetical protein in a murine model of visceral leishmaniasis. Parasite Immunol..

[66] Martins V.T., Chavez-Fumagalli M.A., Lage D.P., Duarte M.C., Garde E., Costa L.E., da Silva V.G., Oliveira J.S., de Magalhaes-Soares D.F., Teixeira S.M.R. (2015). Antigenicity, Immunogenicity and Protective Efficacy of Three Proteins Expressed in the Promastigote and Amastigote Stages of *Leishmania infantum* against Visceral Leishmaniasis. PLoS ONE.

[67] Ramirez L., Santos D.M., Souza A.P., Coelho E.A.F., Barral A., Alonso C., Escutia M.R., Bonay P., de Oliveira C.I., Soto M. (2013). Evaluation of immune responses and analysis of the effect of vaccination of the *Leishmania major* recombinant ribosomal proteins L3 or L5 in two different murine models of cutaneous leishmaniasis. Vaccine.

[68] Costa L.E., Chavez-Fumagalli M.A., Martins V.T., Duarte M.C., Lage D.P., Lima M.I.S., Pereira N.C.D., Soto M., Tavares C.A.P., Goulart L.R. (2015). Phage-fused epitopes from *Leishmania infantum* used as immunogenic vaccines confer partial protection against *Leishmania amazonensis* infection. Parasitology.

[69] Margaroni M., Agallou M., Athanasiou E., Kammona O., Kiparissides C., Gaitanaki C., Karagouni E. (2017). Vaccination with poly (D,L-lactide-co-glycolide) nanoparticles loaded with soluble *Leishmania* antigens and modified with a TNFalpha-mimicking peptide or monophosphoryl lipid A confers protection against experimental visceral leishmaniasis. Int. J. Nanomed..

