# Supplementary material for: Immunization with the HisAK70 DNA Vaccine Induces Resistance against Leishmania Amazonensis Infection in BALB/c Mice
Source: Vaccines (Basel). 2019 Nov 14;7(4):183. doi: 10.3390/vaccines7040183 (PMC6963319; doi:10.3390/vaccines7040183)
Supplement: Supplementary file 1 [file vaccines-07-00183-s001.pdf]

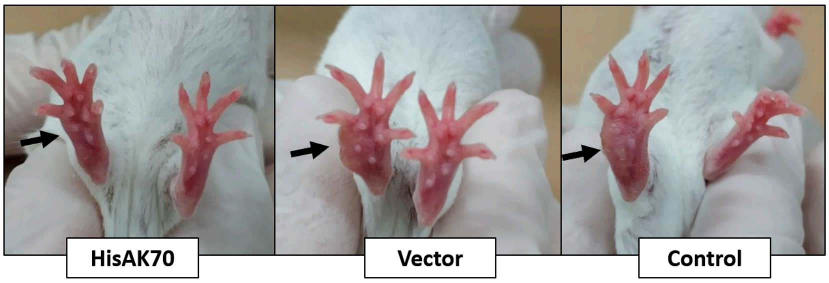

Figure S1. HisAK70 immunization induces protection against *L. amazonensis* infection. Lesion size was measured weekly at the inoculation site during the course of the infection. Data are presented as the mean ± S.D. (n = 5). Images show macroscopically the size of the lesion (indicated by arrows) at twelve weeks post-infection. We can observe the differences between immunized and control mice.
